# Comprehensive dataset of shotgun metagenomes from oxygen stratified freshwater lakes and ponds

**DOI:** 10.1038/s41597-021-00910-1

**Published:** 2021-05-14

**Authors:** Moritz Buck, Sarahi L. Garcia, Leyden Fernandez, Gaëtan Martin, Gustavo A. Martinez-Rodriguez, Jatta Saarenheimo, Jakob Zopfi, Stefan Bertilsson, Sari Peura

**Affiliations:** 1 Department of Aquatic Sciences and Assessment, Swedish University of Agricultural Sciences, Uppsala, Sweden; 2 Department of Ecology, Environment and Plant Sciences, Science for Life Laboratory, Stockholm University, Stockholm, Sweden; 3 Department of Ecology and Genetics, Limnology, Uppsala University, Uppsala, Sweden; 4 Department of Forest Mycology and Plant Pathology, Science for Life Laboratory, Swedish University of Agricultural Sciences, Uppsala, Sweden; 5 Agroenvironmental Science Department, University of Puerto Rico, Mayaguez Campus, San Juan, Puerto Rico USA; 6 Department of Biological and Environmental Science, University of Jyväskylä, Jyväskylä, Finland; 7 Department of Environmental Sciences, Aquatic and Stable Isotope Biogeochemistry, University of Basel, Basel, Switzerland

**Keywords:** Water microbiology, Metagenomics, Freshwater ecology, Microbial ecology

## Abstract

Stratified lakes and ponds featuring steep oxygen gradients are significant net sources of greenhouse gases and hotspots in the carbon cycle. Despite their significant biogeochemical roles, the microbial communities, especially in the oxygen depleted compartments, are poorly known. Here, we present a comprehensive dataset including 267 shotgun metagenomes from 41 stratified lakes and ponds mainly located in the boreal and subarctic regions, but also including one tropical reservoir and one temperate lake. For most lakes and ponds, the data includes a vertical sample set spanning from the oxic surface to the anoxic bottom layer. The majority of the samples were collected during the open water period, but also a total of 29 samples were collected from under the ice. In addition to the metagenomic sequences, the dataset includes environmental variables for the samples, such as oxygen, nutrient and organic carbon concentrations. The dataset is ideal for further exploring the microbial taxonomic and functional diversity in freshwater environments and potential climate change impacts on the functioning of these ecosystems.

## Abstract (summary)

**Table:** 

Measurement(s)	Metagenome
Technology Type(s)	DNA sequencing
Factor Type(s)	lake
Sample Characteristic - Environment	lake with an anoxic hypolimnion • freshwater biome
Sample Characteristic - Location	Sweden • Finland • Canada • Switzerland • Commonwealth of Puerto Rico

Machine-accessible metadata file describing the reported data: 10.6084/m9.figshare.14191043

## Background & Summary

**Fig. 1:**
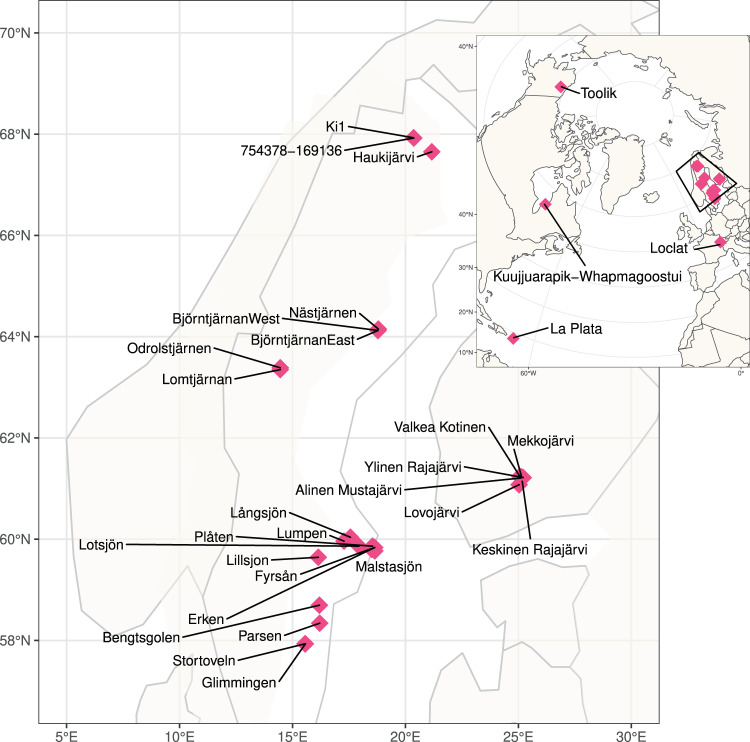
Global distribution of sampling sites. Large Scandinavian map represents the squared region in the insert.

Stratified lakes are a typical feature of the northern landscapes and are also significant sources of greenhouse gas (GHG) emissions^1^. These lakes largely reside in regions critically impacted by climate change^2^ and the future contribution of these lakes to climate change via GHG emissions is dependent on the microorganisms inhabiting their waters^3,4^. In this regard, organisms residing in the anoxic compartment of these waters are of particular interest, as many of the more potent GHGs are produced under such conditions^1^. However, our knowledge regarding the diversity and functioning of these microbial communities is still sparse, and only a few studies have addressed the ecology and functional features of microorganisms in anoxic lake compartments^5–9^. To address this gap in knowledge, we have collected a set of 267 samples from 41 waterbodies including thermally stratified lakes and ponds from boreal and subarctic regions, as well as a depth profile of a tropical reservoir in Puerto Rico and a time series of depth-resolved samples from a temperate and seasonally stratifying eutrophic lake in Switzerland (Fig. 1, Table 1). For the majority of the lakes, samples were available from across the water column, including the oxic epilimnion, the oxygen transition zone (metalimnion) and the deep anoxic hypolimnion (Auxillary Table S1)^10^. Additionally, diverse environmental factors were analysed for all samples, including but not limited to, oxygen, nutrients and organic carbon concentrations (Auxillary Table S1)^10^. For all samples, metagenomes were sequenced using deep shotgun sequencing on the Illumina NovaSeq platform at the Science for Life Laboratory (Uppsala University, Uppsala, Sweden). Additionally, for two of the waterbodies (Alinen Mustajärvi and Lomtjärnan), genomes from single cells were amplified and sequenced, specifically targeting poorly known community members, such as members of the lineage *Chlorobia* and candidate phyla radiation.

**Table 1:** Location, maximum depth, number of samples and sampling years for all of the lakes included to the dataset.

site name	country	latitude	longitude	max depth sampled	number of samples	sampling years
SAS B1_2	Canada	55.23	−77.7	0.45	3	2014; 2017
SAS B4	Canada	55.22	−77.7	NA	2	2014; 2017
SAS C2_4	Canada	55.23	−77.7	0.75	3	2014; 2017
SAS C5	Canada	55.23	−77.7	NA	2	2014; 2017
SAS F5	Canada	55.23	−77.69	2.35	1	2014; 2017
SAS G1	Canada	55.23	−77.7	0.15	2	2014; 2017
SAS H1	Canada	55.23	−77.7	NA	1	2014; 2017
SAS I1_2	Canada	55.23	−77.7	0.35	2	2014; 2017
SAS I3	Canada	55.23	−77.7	NA	2	2014; 2017
SAS2A	Canada	55.23	−77.7	2.25	4	2014; 2017
SAS2B	Canada	55.23	−77.7	2.05	4	2014; 2017
SAS2C	Canada	55.23	−77.7	1.85	4	2014; 2017
SAS2D	Canada	55.23	−77.69	2.35	4	2014; 2017
Alinen Mustajärvi	Finland	61.21	25.11	6.56	42	2014-2015;2018
Keskinen Rajajärvi	Finland	61.22	25.22	10	5	2015
Lovojärvi	Finland	61.08	25.03	13.9	7	2015
Mekkojärvi	Finland	61.23	25.14	3.6	51	2011–2015
Valkea Kotinen	Finland	61.24	25.06	4.5	4	2015
Ylinen Rajajärvi	Finland	61.22	25.21	5	4	2015
La Plata	Puerto Rico	18.33	−66.24	15	8	2018
Bengtsgolen	Sweden	67.92	20.37	5.5	4	2018
Björntjärnan	Sweden	58.7	16.19	0.2	1	2018
Erken	Sweden	64.12	18.78	8	8	2018
Fyrsån	Sweden	59.84	18.64	20	12	2018
Glimmingen	Sweden	59.8	18.51	0.1	1	2018
Haukijärvi	Sweden	57.93	15.57	0.4	1	2018
Kiruna 754378–169136	Sweden	67.65	21.17	3.1	4	2018
Kiruna Ki1	Sweden	67.93	20.36	2.7	4	2018
Långsjön	Sweden	59.64	16.14	0.2	1	2018
Lillsjon	Sweden	63.35	14.46	3.5	21	2018
Lomtjärnan	Sweden	59.86	17.94	0.1	1	2016–2018
Lotsjön	Sweden	59.96	17.28	0.1	1	2018
Lumpen	Sweden	60.04	17.56	0.1	1	2018
Malstasjön	Sweden	59.77	18.64	0.1	1	2018
Nästjärnen	Sweden	64.15	18.8	5	4	2018
Odrolstjärnen	Sweden	63.39	14.46	4.4	2	2018
Parsen	Sweden	58.34	16.2	0.2	1	2018
Plåten	Sweden	59.86	18.54	0.1	1	2018
Stortoveln	Sweden	57.93	15.55	0.2	1	2018
Loclat	Switzerland	47.01	6.59	9	44	2008–2009
Toolik	USA	68.63	−149.6	NA	4	2017

Our goal was to collect a comprehensive dataset that would allow broad analyses of the functioning of the microbial communities in oxygen stratified lakes with emphasis on lakes representing high carbon concentration but with variable environmental conditions with regards to nutrient concentrations, trophic state and other lake features. Based on this data, it is possible to describe the taxonomic identities and genome-encoded functional traits of the predominant microbes across boreal and subarctic lakes and ponds. The dataset represents a major asset for advancing our understanding of the biogeochemical and ecological functioning of these key environments and their present and possible future roles in elemental cycles. The dataset as such can be used, for example, for assessing general metabolic potential of the total lake communities. Further, we have assembled and binned the data into 12665 metagenome-assembled genomes (MAGs), which were further clustered into 3640 metagenomic Operational Taxonomic Units (mOTUs; species level genome clusters). Of these mOTUs, 328 are classified down to species level, while 2141, 878, 220, and 65 could only be assigned to genus, family, order, and class level, respectively. This dataset can, thus, be used for the exploration of population genomes, to study individual community members, and for deeper genome characterization of poorly known members of the resident communities, including their metabolic potentials.

## Methods

### Sample collection

**Fig. 2:**
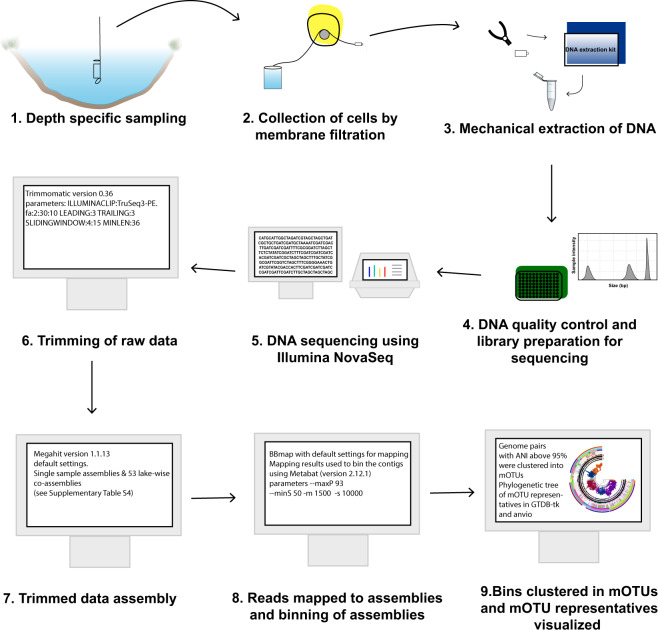
Overview of the workflow from sample collection to mOTUs.

The 267 samples were collected between 2009 and 2018 from 41 locations expanding from the subarctic region to the tropics (Fig. 1, Auxillary Table S1)^10^ and processed using the same analytical pipeline (Fig. 2). The majority of the samples were collected using a depth-discrete Limnos tube-sampler (Limnos, Poland), with the exception of the samples from La Plata reservoir (Puerto Rico), which were collected using horizontal Van Dorn sampler (5 L capacity) and samples from Lake Loclat, which were collected using a deployed PVC-inlet connected to a peristaltic pump via tubing. Of all the lakes, 29 were sampled during the open water season and the majority of the lakes were sampled once. For 12 of the lakes only surface samples taken during the ice-covered period in winter were available, and one of the Swedish lakes (Lake Lomtjärnan) was sampled twice during the ice-covered period. Moreover, a total of 5 samples (one depth profile) from the time series of the Swiss lake (Loclat) were taken from under the ice. Time series samples were taken for Lake Loclat (seven time points, Auxillary Table S1)^10^ and for Lake Mekkojärvi (22 time points, see Saarenheimo *et al*.^11^ for details). For most lakes and ponds, samples were collected from multiple depths, including samples from the oxic surface layer (epilimnion), the layer with steepest change in oxygen concentration and temperature (metalimnion) and from the layer where oxygen levels were below the detection limit (hypolimnion). The exception to this were the 12 Swedish lakes sampled during ice-covered period, and five shallow ponds in Canada, for which only one sample from the oxic surface layer was taken (see Auxillary Table S1)^10^.

From two of the lakes, Lake Lomtjärnan in Sweden and Lake Alinen Mustajärvi in Finland, samples were collected also for single cell sorting. From both locations samples were preserved in glycerol-TE (gly-TE) and from Lomtjärnan samples were preserved also using phosphate buffered saline (PBS). For both preservants, the samples were flash frozen in liquid nitrogen after first incubating for 1 minute at ambient temperature.

Simultaneous to collection of the DNA samples, also samples for environmental variables were taken. Variables included temperature, pH, conductivity, oxygen, total and dissolved nutrients (P and N species), gases (CO_2_ or dissolved inorganic carbon and methane (CH_4_)), total or dissolved organic carbon, iron, sulfate and chlorophyll a (Auxillary Table S1 and Auxillary Table S2^10^ for the methods). As the samples were collected during multiple years and by different research groups, there was some variation for the procedures between the different sampling occasions, leading to variation in the final set of environmental data across the samples.

### DNA extraction and metagenome sequencing

Most of the DNA samples were collected on 0.2 µm Sterivex filters (Millipore), except for the time-series samples collected from Loclat, which were collected by vacuum filtration onto 47 mm polycarbonate membrane filters with 0.2 μm pore size, and time series samples from Finnish Lake Mekkojärvi, for which the water for DNA extraction was collected from epilimnion (0–0.5 m), metalimnion (0.5–1 m) and hypolimnion (1–3 m) and pooled samples from each stratum were stored in 100 ml plastic containers and frozen at −20 °C and eventually freeze-dried (Alpha 1–4 LD plus, Christ). For all filter samples, water was filtered until the filter clogged. All filters were stored frozen (−20 to −80 °C) until the extraction of DNA. For all samples, DNA was extracted using PowerSoil DNA extraction kit (MoBio, Carlsbad, CA, USA) following the manufacturer’s instructions and the DNA concentrations were measured using Qubit dsDNA HS kit (Thermo Fisher Scientific Inc.).

Sequencing libraries were prepared from 10 or 20 ng of DNA using the ThruPLEX DNA-seq Prep Kit according to the manufacturer’s preparation guide. Briefly, the DNA was fragmented using a Covaris E220 system, aiming at 400 bp fragments. The ends of the fragments were end-repaired and stem-loop adapters were ligated to the 5′ ends of the fragments. The 3′ end of the stem loop were subsequently extended to close the nick. Finally, the fragments were amplified and unique index sequences were introduced using 7 cycles of PCR followed by purification using AMPure XP beads (Beckman Coulter).

The quality of the libraries was evaluated using the Agilent Fragment Analyzer system and the DNF-910-kit. The adapter-ligated fragments were quantified by qPCR using the Library quantification kit for Illumina (KAPA Biosystems/Roche) on a CFX384Touch instrument (BioRad) prior to cluster generation and sequencing.

The sequencing libraries were pooled and subjected to cluster generation and paired-end sequencing with 150 bp read length S2/S4 flow-cells and the NovaSeq 6000 system (Illumina Inc.) using the v1 chemistry according to the manufacturer’s protocols. Negative controls were included to the sequencing as well as 1% of PhiX control library as a positive control.

Base calling was done on the instrument by RTA (v3.3.3, 3.3.5, 3.4.4) and the resulting.bcl files were demultiplexed and converted to fastq format with tools provided by Illumina Inc., allowing for one mismatch in the index sequence. Additional statistics on sequence quality were compiled with an in-house script from the fastq-files, RTA and CASAVA output files. Sequencing was performed by the SNP&SEQ Technology Platform in Uppsala, Sweden.

### Single-cell sorting and DNA amplification

All Gly-TE cryopreserved samples were thawed and diluted in 1 xPBS if needed while all plates with PBS were UV-treated with a dose of 2 J prior to sorting. Samples collected from both lakes were sorted, and then screened for organisms belonging to candidate phyla radiation. Samples collected from Lake Lomtjärnan were additionally subjected to sorting based on autofluorescence to identify and sequence cells belonging to lineage *Chlorobia*.

For obtaining SAGs from representatives of the candidate phyla radiation (CPR), samples were first stained with 1 x SYBR Green I for approximate 30 minutes. Subsequent single cell sorting was performed with a MoFlo Astrios EQ (Beckman Coulter, USA) cell sorter using a 488 nm laser for excitation, 70 µm nozzle, sheath pressure of 60 psi and 0.1 µm sterile filtered 1x PBS as sheath fluid. Individual cells were deposited into empty 384-well plates (Biorad, CA USA) UVed at 2 Joules using a CyCloneTM robotic arm and the most stringent single cell sort settings (single mode, 0.5 drop envelope). Green fluorescence (488–530/40) was used as trigger and sort decisions were made based on combined gates of 488–530/40 Height log vs 488–530/40 Area log and 488–530/40 Height log vs SSC with increasing side scatter divided up in three different regions. Flow sorting data was interpreted and displayed using the associated software Summit v 6.3.1. Next, individual cells were subject to lysis, neutralization and whole genome amplification using MDA based on the protocol and workflow described by Rinke *et al*.^12^ but with several modifications. Reagent mastermixes were added using the MANTIS liquid dispenser (Formulatrix) and the LV or HV silicone chips. The lysozyme, D2 buffer, stop solution and MDA-mastermix were each dispensed with its own chip. Most MDA-reactions were run using the phi29 from ThermoFisher but a few were run with a more heat-stable phi29, EquiPhi also provided by ThermoFisher. The MDA reaction was carried out in a total volume of 5.2 µl. Thawed, sorted cells were first pre-treated with 400 nl/well of 12 U/µl of Ready-Lyse™ Lysozyme Solution (R1804M, Lucigen) at room temperature for 15 minutes before adding 400 nl Qiagen lysis buffer D2 followed by incubation at 95 °C for 10 seconds and 10 minutes on ice. Reactions were neutralized by adding 400 nl Qiagen Stop solution. Four µl of MDA mix containing 1x reaction buffer, 0.4 mM dNTP, 0.05 mM exonuclease-resistant Hexamers, 10 mM DTT, 1.7 U phi29 DNA polymerase (ThermoFisher Scientific) and 0.5 µM Syto13 was added to a final reaction volume of 5.2 µl. All reagents except SYTO13 were UV decontaminated at 2 Joules in a UV crosslinker. The whole genome amplification was run at 30 °C for 7 or 10 h followed by an inactivation step at 65 °C for 5 min. The reaction was monitored in real time by detection of SYTO13 fluorescence every 15 minutes using a FLUOstar® Omega plate reader (BMG Labtech, Germany) or a qPCR instrument. The EquiPhi protocol was run as previously described for ThermoFisher phi29 with the following exceptions; the EquiPhi polymerase was added in 1U/reaction, reaction buffer included with the polymerase was used and the reaction was carried out at 45 °C. The single amplified genome (SAG) DNA was stored at −20 °C until further PCR screening, library preparation and Illumina sequencing.

The CPR SAGs were screened using the bacterial PCR primers targeting the 16 S rRNA gene, Bact_341 F and Bact_805 R^13^. The reactions were run in a LightCycler 480 PCR machine (ROCHE, MA USA) in 10 µl and a final concentration of 1 x LightCycler480 SYBR Green I Master mix, 0.25 µM of each primer and 2 µl of 60 to 80 times diluted SAGs. Following a 3 min denaturation at 95 °C, targets were amplified for 40 cycles of 95 °C for 10 s, 55 °C for 20 s, 72 °C for 30 s and a final 10 min extension at 72 °C followed by melting curve analysis. The products were purified using the NucleoSpin Gel and PCR clean-up purification kit (Macherey-Nagel, Germany), quantified using the Quant-iT TM PicoGreen® dsDNA assay kit (Invitrogen, MA USA) in a FLUOstar® Omega microplate reader (BMG Labtech, Germany) and submitted for identification by Sanger sequencing at Eurofin Genomics. All SAGs were further screened using the newly designed primers targeting the phylum Parcubacteria 684F-OD1 (3′ GTAGKRRTRAAATSCGTT 5′) and 784 R (5′ TAMNVGGGTATCTAATCC -3′). These primers target with good specificity 67% of Parcubacteria in the SILVA database^14^. Parcu-PCR was run at 3 min at 95 °C, 40 cycles of 95 °C for 10 s, 55 °C for 20 s, 72 °C for 30 s and a final 10 min extension at 72 °C followed by melting curve analysis. The products were purified using the NucleoSpin Gel and PCR clean-up purification kit (Macherey-Nagel, Germany), quantified using the Quant-iT TM PicoGreen® dsDNA assay kit (Invitrogen, MA USA) in a FLUOstar® Omega microplate reader (BMG Labtech, Germany) and submitted for identification by Sanger sequencing at Eurofin Genomics.

To recover *Chlorobia* single amplified genomes, sorting was done in 2016 on a MoFlo™ Astrios EQ sorter (Beckman Coulter, USA) using a 488 and 532 nm laser for excitation, a 70 μm nozzle, a sheath pressure of 60 psi, and 0.1 μm filtered 1x PBS as sheath fluid. An ND filter ND = 1 and the masks M1 and M2 were used. The trigger channel was set to the forward scatter (FSC) at a threshold of 0.025% and sort regions were defined on autofluorescence using laser 532 nm and band pass filters 710/45 and 664/22. Three populations were sorted based on differences in autofluorescence signals. The sort mode was set to single cell with a drop envelope of 0.5. The target populations were sorted at approximately 400 events per second into 96-well plates containing 1 µl 1x PBS per well with either 1 or 10 cells (positive control) deposited. A few wells remained empty (no cell sorted) were kept as negative controls. Sorted plates were stored frozen at −80 °C.

The subsequent whole genome amplification was performed in 2018 using the REPLI-g Single Cell kit (QIAGEN) following the instructions provided by the manufacturer but with total reaction volume reduced to 12.5 µl. The denaturation reagent D2, stop solution, water, and reagent tubes and strips were UV-treated at 2.5 J. The lysis was changed slightly to 10 min at 65 °C, followed by 5 min on ice before adding the stop solution. To the master mix containing water, reaction buffer, and the DNA the polymerase we added SYTO 13 (Invitrogen) at a final concentration of 0.5 µM. The amplification was performed at 30 °C for 8 hours in a plate reader with fluorescence readings every 15 min. The reaction was stopped by incubating it for 5 min at 65 °C. The plate was stored for less than a week at −20 °C. Amplified DNA was mixed thoroughly by pipetting up and down 20 times before diluting it 50x and 100x in nuclease-free water. The DNA was screened for bacterial 16 S rRNA applying the primers Bact_341 F (5′- CCTACGGGNGGCWGCAG- 3′) and Bact_805 R (5′- GACTACHVGGGTATCTAATCC-3′)^13^ using the LightCycler® 480 SYBR Green I Master (Roche) kit. The PCR mix contained 1.5 µl diluted amplified DNA, 1x the LightCycler® 480 SYBR Green I Master mix, 0.25 µM of each primer, and nuclease-free water in a total reaction volume of 10 µl. The PCR cycling (5 min at 95 °C, followed by 40 cycles of 10 sec at 95 °C, 20 sec at 60 °C, 30 sec at 72 °C) was followed by meltcurve analysis on the LightCycler® 480 Instrument (Roche). DNA of confirmed *Chlorobia* was sent to sequencing as outlined below.

### Library preparation and Illumina sequencing of the single cells

For the CPR-targeted analysis, Illumina libraries were prepared from sixty SAGs mainly selected from the screening procedure in a PCR-free workflow using the sparQ DNA Frag & Library Prep Kit (Quantabio) and IDT for Illumina TruSeq UD Indexes (Illumina). Libraries were prepared from 50–250 ng of MDA-products in 25% of the recommended reaction volumes according to manufacturer’s instructions. The MDA-products were fragmented for 7 minutes (5 minutes for 4 samples) without using the DNA Frag Enhancer Solution. Library insert sizes were determined using Bioanalyzer High Sensitivity DNA Kit (Agilent). Each library was quantified using the KAPA Library Quantification kit (Roche) in 5 µl reaction volumes in a 384-well plate run on LightCycler 480 (Roche) to allow equimolar pooling before sequencing on Illumina HiSeqX v2.5 PE 2 × 150 bp including negative and positive (PhiX) controls.

For the *Chlorobia*-targeted sequencing, amplified DNA from 23 SAGs were quantified individually with Qubit dsDNA HS assay kit (ThermoFisher Scientific) and diluted to 0.2 ng/ul in nuclease free water. Sequencing libraries were prepared with Nextera XT DNA Library Preparation Kit and combinatorial combinations of molecular identifiers in the Nextera XT Index Kit (Illumina, CA USA) according to manufacturer’s instructions. Libraries with an average length of 1200 bp were quantified with Qubit dsDNA HS assay kit to allow pooling of equal amounts of the libraries based on mass. The libraries were sequenced on an Illumina MiSeq v3 PE 2 × 300 bp including negative and positive (PhiX) controls.

### Data processing of the metagenome and single cell sequences

The metagenome sequencing resulted in a total of ~10^7^ paired-end reads of length 2 × 150 bp, amounting to a total of total 3 Tbp. The raw data was trimmed using Trimmomatic (version 0.36; parameters: ILLUMINACLIP:TruSeq 3-PE.fa:2:30:10 LEADING:3 TRAILING:3 SLIDINGWINDOW:4:15 MINLEN:36)^15^ (Auxillary Table S3)^10^. The trimmed data was assembled using Megahit (version 1.1.13)^16^ with default settings. Two types of assemblies were done, single sample assemblies for all the samples individually and a total of 53, mainly lake-wise, co-assemblies (see Auxillary Table S4)^10^, some samples of the Canadian ponds have also been coassembled with previously sequenced libraries of the same sample (see Auxillary Table S5)^10^. The relevant quality controlled reads were mapped to all the assemblies using BBmap^17^ with default settings and the mapping results were used to bin the contigs using Metabat (version 2.12.1, parameters --maxP 93 --minS 50 -m 1500 -s 10000)^18^. Genes of obtained bins were predicted and annotated using Prokka (version 1.13.3)^19^ using standard parameters except for the bin containing all the unbinned contigs where the –metagenome flag was used. Single-cell libraries were processed similarly to the metagenomes, but without the binning step, and using the single-cell variant of the SPAdes^20^ assembler instead of Megahit.

Prokaryotic completeness and redundancy of all bins from Metabat and for all assembled single cells were computed using CheckM (version 1.0.13)^21^ (Auxillary Tables S6 and S7 for MAGs and SAGs, respectively)^10^. Average Nucleotide Identity (ANI) for all bin-pairs was computed with fastANI (version 1.3)^22^. The bins were clustered into metagenomic Operational Taxonomic Units (mOTUs) starting with 40% complete genomes with less than 5% contamination. Genome pairs with ANI above 95% were clustered into connected components. Additionally, less complete genomes were recruited to the mOTU if its ANI similarity was above 95%. Bins were taxonomically annotated in a two-step process. GTDB-Tk (version 102 with database release 89)^23^ was used first with default settings. Using this classification an lca database for SourMASH (version 1.0)^24^ was made. This database as well as one based on the GTDB release 89 was then used with SourMASH’s lca classifier for a second round of classification of bins that were not annotated with GTDB-tk (Auxillary Table S8)^10^.

**Fig. 3:**
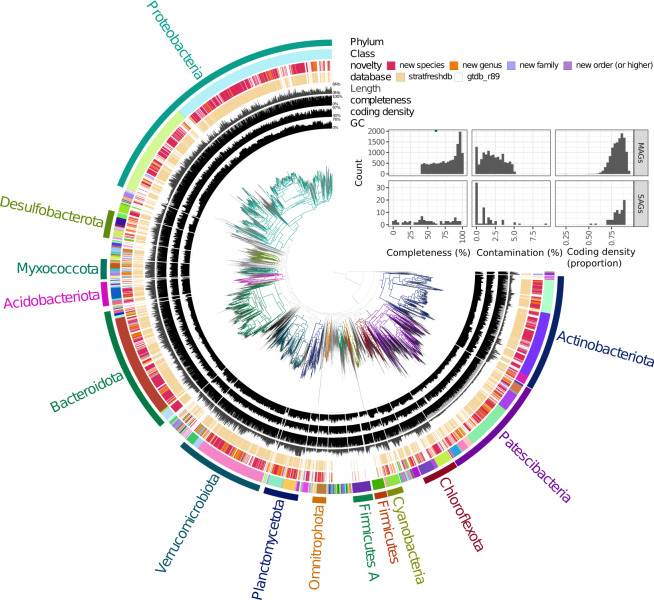
Bacterial diversity of the stratfreshDB^27^. The insert illustrates the quality of the MAGs and SAGs included in the tree. Interactive version of the tree with more information available at https://anvi-server.org/moritzbuck/bacterial_diversity_of_the_stratfreshdb.

**Fig. 4:**
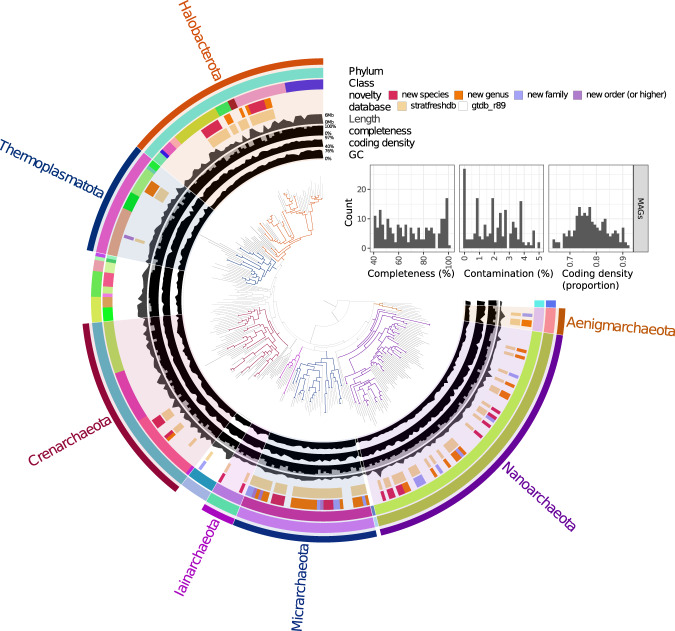
Archaeal diversity of the stratfreshDB^27^. The insert illustrates the quality of the MAGs included in the tree. Interactive version of the tree with more information available at https://anvi-server.org/moritzbuck/archaeal_diversity_of_the_stratfreshdb.

The taxonomic diversity of the bacterial (Fig. 3) and archaeal (Fig. 4) mOTUs, respectively, were visualized in a tree format. The trees were computed using GTDB-tk with one representative MAG per mOTU of the stratfreshDB, and one random representative genome per family of the GTDB. Trees were visualized using anvi’o^25^.

## Data Records

All sequences are deposited to the European Nucleotide Archive (ENA, mirrored to SRA, and accessible at the NCBI) under the project number PRJEB38681^26^. Statistics for reads, metagenome assemblies, high-quality bins (e.g. MAGs), and SAGs can be found in Auxillary Table S3, S2, S4, and S5^10^, respectively. For the MAGs the accession numbers are found in Auxillary Table S3 and the for the SAGs in Auxillary Table S7^10^. Additional tables and information can be found under the 10.17044/scilifelab.13005311.v2^27^.

## Technical Validation

**Fig. 5:**
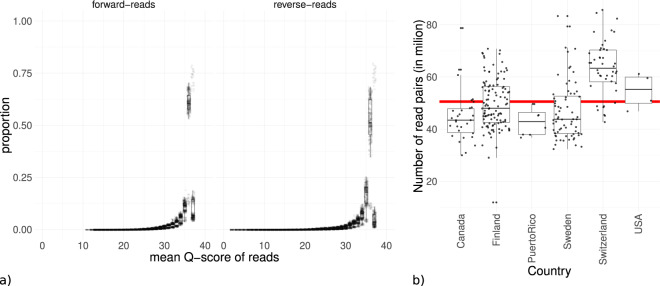
Overview of quality of metagenomic libraries. (**a**) boxplot summarizing read-quality histograms of all libraries, each dot represents the proportion of reads of a specific library with a mean phred-score of that value. (**b**) boxplots of metagenome depths across regions, each dot represents the number of read pairs for a specific library. The readline is the mean number of pairs for all libraries. Full data available in Auxillary Data S9^10^.

The Q scores for the raw reads were calculated prior to any further processing using the MultiQC^25^ wrapper for FastQC^28^ (Fig. 5). The observed quality distribution showed that most of the reads had Q scores Q30 or higher indicating libraries with low error rates and without serious abnormalities (Fig. 5a). Number of reads per sequenced library was relatively even also indicating no quality problems (Fig. 5b). Full data of the quality reports can be found in Auxillary Data S9^10^, including machine-readable quantitative data, the summarized data of the figure and HTML-report. The quality of the MAGs and SAGs was computed with CheckM^21^ (Figs. 3 and 4, Auxillary Tables S6 and S7^10^). The MAGs and SAGs were deemed as high quality if the completeness was >70%, and contamination was <5% and moderate quality if the completeness was >40%, and contamination was <5%. Using these thresholds, the data had about 8500 high quality and 4500 moderate quality MAGs (Table 2).

**Table 2:** Summary of all genomic data.

Type of data	Number	Total size (Gbases)
Libraries (samples)	346	2800
Assembled metagenomes	341	105
Total number of bins	~500000	98
Number of high quality metagenome assembled genomes (MAGs; >70% complete, <5% contaminated) from the total number of bins	8554	26
Number of moderate quality metagenome assembled genomes (MAGs; >40% complete, <5% contaminated) from the total number of bins	4487	7
Single-amplified genomes (SAGs)	83	0.1

## Usage Notes

The full metagenome samples can be accessed at ENA^26^. Additionally, all bins and SAGs as well as subsets of reads for all samples (1 M reads per sample) can be accessed at https://export.uppmax.uu.se/uppstore2018116/stratfreshdb/ using the paths in the files available at 10.17044/scilifelab.13005311.v2^27^ for navigation.

## Data Availability

Code used to process the data is available at https://github.com/moritzbuck/metasssnake, code for the computing of mOTUs is available at https://github.com/moritzbuck/mOTUlizer and some additional scripts, particularly the script used for the submission of the data and summary of the quality reports is available at https://github.com/moritzbuck/0023_anoxicencyclo.
